# Crystallographic characterization of (C_5_H_4_SiMe_3_)_3_U(BH_4_)

**DOI:** 10.1107/S2056989021002425

**Published:** 2021-03-12

**Authors:** Cory J. Windorff, Justin N. Cross, Brian L. Scott, Stosh A. Kozimor, William J. Evans

**Affiliations:** a Los Alamos National Laboratory, Los Alamos, New Mexico 87544, USA; b Department of Chemistry, University of California, Irvine, California 92697, USA

**Keywords:** crystal structure, uranium, borohydride, cyclo­penta­dienyl compounds

## Abstract

New syntheses have been developed for the synthesis of (borohydrido-κ^3^ *H*)tris­[η^5^-(tri­methyl­sil­yl)cyclo­penta­dien­yl]uranium(IV), [U(BH_4_)(C_8_H_13_Si)_3_] or Cp′_3_U(BH_4_) (Cp′ = C_5_H_4_SiMe_3_) and its structure has been determined by single-crystal X-ray crystallography. This compound crystallized in the space group *P*  and the structure features three *η* ^5^-coordinated Cp′ rings and a *κ* ^3^-coordinated (BH_4_)^−^ ligand.

## Abstract 2

The structure of Cp′_3_U(BH_4_), (C_5_H_4_SiMe_3_)_3_U(BH_4_), at 112 K has triclinic (*P* 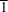) symmetry. It is of inter­est with respect to borohydride coordination modes.

## Chemical context   

Actinide borohydrides have been of inter­est since the 1940s, owing to their potential volatility and applied use in vapor deposition technologies for the production of thin films (Hoekstra & Katz, 1949 ▸; Daly & Girolami, 2010 ▸). Uranium borohydride compounds are structurally inter­esting because the (BH_4_)^−^ ligand can coordinate large electropositive cations (such as uranium) in several modes. For example, *κ* ^1^, *κ* ^2^, and *κ* ^3^ U–(BH_4_) binding has previously been reported (Ephritikhine, 1997 ▸). Borohydrides can also achieve high coordination numbers with uranium, *e.g*. the oligomeric 14-coordinate U(BH_4_)_4_ (Bernstein *et al.*, 1972 ▸). Although several cyclo­penta­dienyl uranium borohydrides have been crystallographically characterized (Ephritikhine, 1997 ▸), the structure of Cp′_3_U(BH_4_) (Cp′ = C_5_H_4_SiMe_3_), made in 1992 (Berthet & Ephritikhine, 1992 ▸), has not been reported. Our inter­est in Cp′ uranium chemistry (MacDonald *et al.*, 2013 ▸; Windorff *et al.*, 2017 ▸) prompted us to determine the coordination mode of (BH_4_)^−^ within the tris-cyclo­penta­dienyl uranium platform using single-crystal X-ray diffraction. Toward this end, we developed new synthetic routes to the Cp′_3_U(BH_4_) compound.
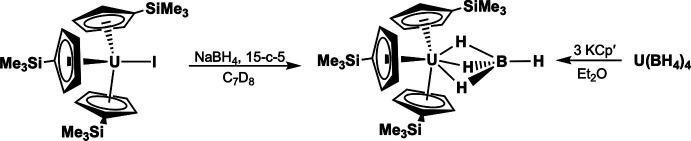


The Cp′_3_U(BH_4_) compound was originally synthesized by reacting Cp′_3_UH with H_3_B-PPh_3_ (Berthet & Ephritikhine, 1992 ▸). Our attempts to repeat this procedure in toluene and diethyl ether solvents were unsuccessful, potentially because we were uncertain about the details of the reaction. However, we were successful in synthesizing Cp′_3_U(BH_4_) from Cp′_3_UH with H_3_B-PPh_3_ in hot THF solvent. We also observed Cp′_3_U(BH_4_) could be prepared in high yield (96%) by reacting Cp′_3_UI with NaBH_4_ in the presence of 15-crown-5. When this reaction was carried out in toluene at room temperature, the I^−^ ligand was substituted by the (BH_4_)^−^ anion. Another method we developed for synthesizing Cp′_3_U(BH_4_) involved reacting U(BH_4_)_4_ with KCp′ (3 equiv.) in diethyl ether. This reaction, where (BH_4_)^−^ was substituted by (Cp′)^−^, also proceeded in high yield (89%). X-ray quality crystals of Cp′_3_U(BH_4_) formed at 253 K overnight from diethyl ether solutions.

Of our two synthetic routes, we preferred making Cp′_3_U(BH_4_) from Cp′_3_UI over U(BH_4_)_4_ because the U(BH_4_)_4_ starting material was more challenging to isolate in a chem­ic­ally pure form. Another inter­esting comparison between the two synthetic methods involved the substitution chemistry. The (Cp′)^−^ anion displaced (BH_4_)^−^ from U(BH_4_)_4_ and (BH_4_)^−^ displaced I^−^ in Cp′_3_UI. Hence, we qualitatively concluded that the stability of the U—*X* bond for mol­ecular compounds dissolved in organic solvents was largest for (Cp′)^−^, inter­mediate for (BH_4_)^−^, and lowest for I^−^. The generality of this conclusion is limited, and we acknowledge the solubility of the other reaction products (such as NaI) might significantly influence the substitution chemistry on uranium.

## Structural commentary   

**Figure 1:**
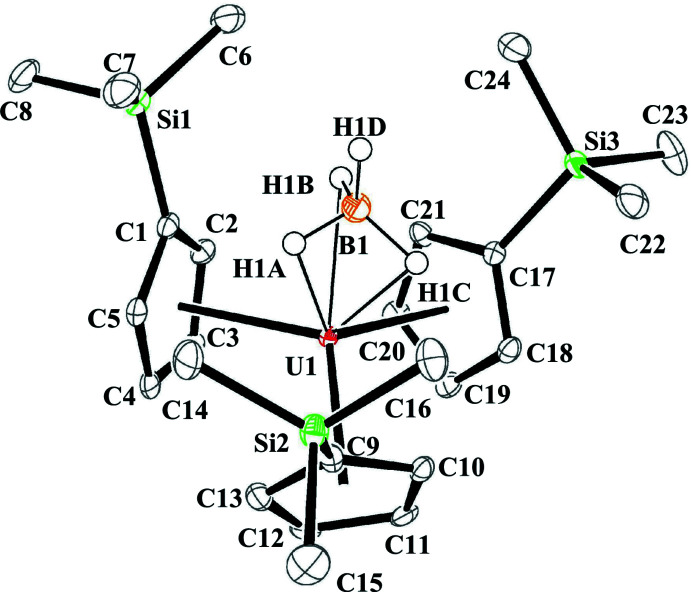
Structure of Cp′_3_U(BH_4_) with atomic displacement parameters drawn at the 50% probability level. Boron-bound hydrogen atoms are represented as isotropic circles. All carbon-bound hydrogen atoms are omitted. Selected structural metrics, U—(Cp′ centroid) average 2.48 (2) Å, U—H average 2.35 (1) Å, (Cp′ centroid)—U—(Cp′ centroid) average 113.9 (6)°, (Cp′ centroid)—U—B average 104.4 (4)°, and terminal B—H distance of 1.11 (5) Å.

Single crystal X-ray data from Cp′_3_U(BH_4_) were refined in the triclinic *P* 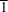 space group with one crystallographically unique mol­ecule in the unit cell, see Fig. 1 ▸. The data are of high quality, and electron-density difference peaks consistent with the location and geometry of bridging hydrides were located from a difference-Fourier map with U—H distances of 2.35 (5), 2.35 (5), and 2.36 (5) Å. Although the uncertainty associated with the U—H bonds is relatively high, they are consistent with previously reported bond lengths for actin­ide(IV) hydride inter­actions (Ephritikhine, 1997 ▸; Daly *et al.*, 2010 ▸). Significantly lower uncertainty is associated with the U—B distance at 2.568 (4) Å, which is similar to two of the three U—B distances in [U(BH_4_)_3_(DME)]_2_(*μ*-O) (DME = 1,2-di­meth­oxy­ethane), 2.574 (6), 2.584 (6), and 2.635 (7) Å (Daly *et al.*, 2012 ▸). The U—B distance in (C_5_H_5_)_3_U(BH_4_) was reported to be 2.48 Å (Zanella *et al.*, 1988 ▸), although disorder in that structure prevented a full solution from being obtained. Theoretical calculations on (C_5_H_5_)_3_U(BH_4_) in the gas phase and in solution predicted U—B distances of 2.533 and 2.557 Å (Elkechai *et al.*, 2009 ▸), which are also consistent with our data. Other (C_5_ *R*_5_)_2_U(BH_4_)_2_ structures showed similar U—B distances of 2.56 (1) Å for [C_5_H_3_(SiMe_3_)_2_]_2_U(BH_4_)_2_ (Blake *et al.*, 1995 ▸), 2.58 (3) Å in (C_5_Me_5_)_2_U(BH_4_)_2_ (Gradoz *et al.*, 1994 ▸, Marsh *et al.*, 2002 ▸), and 2.553 (1) Å in (PC_4_Me_4_)_2_U(BH_4_)_2_ (Baudry *et al.*, 1990 ▸).

The uranium–(Cp′ centroid) distances in Cp′_3_U(BH_4_) range from 2.458–2.500 Å and average 2.48 (2) Å (uncertainty reported as the standard deviation from the mean at 1σ). These uranium–(Cp′ centroid) distances compare well with the 2.473 Å analogous metric in Cp′_3_UCl (Windorff *et al.*, 2017 ▸) and other Cp′_3_U*X* structures (see Table 1 ▸) with average U–(Cp′ centroid) distances of 2.478 (3) Å in Cp′_3_UI (Windorff *et al.*, 2017 ▸), 2.484 (4) Å in Cp′_3_U(*η* ^1^-CH=CH_2_) (Schock *et al.*, 1988 ▸) and 2.478 (7) Å in Cp′_3_U[Si(SiMe_3_)_3_] (Réant *et al.*, 2020 ▸). The 113.9 (6)° average of (Cp′ centroid)—U—(Cp′ centroid) angles in Cp′_3_U(BH_4_) is more acute than the 117.0° angle in Cp′_3_UCl and other Cp′_3_U*X* structures, where the average (Cp′ centroid)—U—(Cp′ centroid) angles were reported as 117 (1)° in Cp′_3_UI, 112 (2)° in Cp′_3_U(*η* ^1^-CH=CH_2_), and 118.7 (4)° in Cp′_3_U[Si(SiMe_3_)_3_]. The more acute (Cp′ centroid)—U—(Cp′ centroid) angles are complemented by a more obtuse average (Cp′ centroid)—U—B angle of 104.4 (4)° in Cp′_3_U(BH_4_), likely due to the close proximity of the (BH_4_)^1−^ ligand compared with (Cp′ centroid)—U—*X* angles of 100.0° in Cp′_3_UCl, 100 (2)° in Cp′_3_UI, 98 (3)° in Cp′_3_U(*η* ^1^-CH=CH_2_), and 96.7 (9)° in Cp′_3_U[Si(SiMe_3_)_3_], see Table 1 ▸.

**Figure 2:**
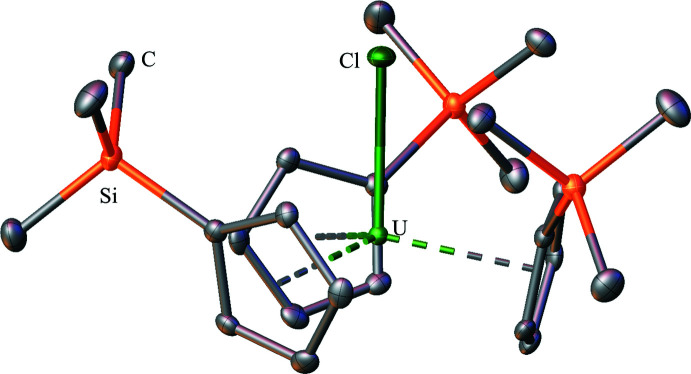
Structure of Cp′_3_UCl with atomic displacement parameters drawn at the 50% probability level, as reproduced from the published CIF (Windorff *et al.*, 2017 ▸); the isomorphous thorium complex, Cp′_3_ThCl, is also known (Réant *et al.*, 2020 ▸). Hydrogen atoms are omitted for clarity.

An unusual feature of the Cp′_3_U(BH_4_) structure is that all three of the tri­methyl­silyl groups are oriented in a single direction towards the (BH_4_)^−^ unit. This orientation has not been observed in other Cp′_3_U(anion) and Cp′_3_U(*μ*-dianion)UCp′_3_ structures, which are shown in Figs. 2 ▸–12 ▸ ▸ ▸ ▸ ▸ ▸ ▸ ▸ ▸ ▸. The closest comparison is with the Cp′_3_UCl structure (Windorff *et al.*, 2017 ▸), where all three tri­methyl­silyl groups are oriented towards the Cl^−^ unit, but twisted down and away from the chloride towards the meridian. The Cp′_3_UI (Windorff *et al.*, 2017 ▸) and Cp′_3_U(*η* ^1^-CH=CH_2_) (Schock *et al.*, 1988 ▸) complexes have one tri­methyl­silyl group pointed away from the anionic ligand. The Cp′_3_U[Si(SiMe_3_)_3_] complex (Réant *et al.*, 2020 ▸) represents the opposite extreme where all of the tri­methyl­silyl groups are oriented away from the [Si(SiMe_3_)_3_]^1−^ unit. Since Cp′_3_U(BH_4_) has the smallest mono-anion of the Cp′_3_U(anion) complexes and the correspondingly smallest (Cp′ centroid)—U—(Cp′ centroid), and the largest (Cp′ centroid)—U—*X* angles, the orientation of the silyl groups could occur due to steric factors. However, it is also possible that some dispersion forces between the (BH_4_)^−^ and the tri­methyl­silyl groups could contribute to the orientation (Liptrot *et al.*, 2016 ▸). It is inter­esting to note that in the Cp′_3_Th*X* series where *X* = Cl (Réant *et al.*, 2020 ▸), Br (Windorff *et al.*, 2017 ▸), and CH_3_ (Wedal *et al.*, 2019 ▸), all three tri­methyl­silyl groups are oriented towards the anion, but twisted down and away from the anion towards the meridian as in Cp′_3_UCl.

## Supra­molecular features   

There are no major supra­molecular features to report. The mol­ecules pack in an alternating 180° rotation from one another within the unit cell and stack ‘head to tail’ between the unit cells.

## Database survey   

A search using the Cambridge Structural Database (Version 5.41, March 2020; Groom *et al.*, 2016 ▸) for borohydride structures containing *η* ^5^-aromatic five-membered rings bound to uranium showed two classes of complexes. There were the uranium(IV) piano-stool complexes: (C_5_H_5_)U(BH_4_)_3_ (DEKVEU and DEKVEU10; Baudry *et al.*, 1985 ▸, 1989 ▸); (C_5_Me_5_)U(BH_4_)(SPS^Me^) (JOJTIM; Arliguie *et al.*, 2008 ▸), where SPS^Me^ = PC_5_H-3,5-Ph,-2,6-(P(S)Ph_2_)-1-Me, a *λ* ^4^-phosphinine with two lateral phosphino­sulfide groups, and the tetra­methyl­phosphol (PC_4_Me_4_) compound (PC_4_Me_4_)(C_8_H_8_)U(BH_4_)(THF) (MOBVEE; Cendrowski-Guillaume *et al.*, 2002 ▸). There were also uranium(IV) metallocene structures, (Ring)_2_U(BH_4_)_2_, where Ring = C_5_H_5_ (CPURBH; Zanella *et al.*, 1977 ▸), C_5_H_3_(SiMe_3_)_2_ (ZEYZOS; Blake *et al.*, 1995 ▸), C_5_Me_5_ (WIFFOG and WIFFOG01; Gradoz *et al.*, 1994 ▸; Marsh *et al.*, 2002 ▸), C_9_H_7_ (VASVUG, C_9_H_7_ = indenide; Rebizant *et al.*, 1989 ▸) and PC_4_Me_4_ (KIJBEK, PC_4_Me_4_ = tetra­methyl­phosphol; Baudry *et al.*, 1990 ▸). The macrocyclic *trans*-calix[2]benzene­[2]pyrrolide (*L*) complex [*L*U(BH_4_)][B(C_6_F_5_)_4_] was also in the database (CUJMEB; Arnold *et al.*, 2015 ▸). This last compound features two *η* ^5^-bound NC_4_H_2_ *R*_2_ ligands. Also in the database were a few examples of uran­ium(III) borohydrides, such as the mono borohydride [(PC_4_Me_4_)_2_U(BH_4_)]_2_ (YEZJES; Gradoz *et al.*, 1994 ▸) and the mixed oxidation state piano stool [Na(THF)_6_][(C_5_Me_5_)U(BH_4_)_3_]_2_ (VAXMUC; Ryan *et al.*, 1989 ▸)] complexes.

There are also three dimeric uranium(IV) complexes with Cp′^−^ ligands, all of the form (Cp′_3_U)_2_(*μ*-*X*) where *X* = O^2−^ (SOSXON; Berthet *et al.*, 1991 ▸), (pyrazine)^2−^, (N_2_C_4_H_4_)^2−^ (EYERIJ; Mehdoui *et al.*, 2004 ▸), and CCO^2−^ (PIKFAT; Tsoureas & Cloke, 2018 ▸). There is also the tetra­metallic (Cp′_3_U)_4_(*μ*-*L*) (PIKDUL; Tsoureas & Cloke, 2018 ▸) where *L* is a complex organic structure containing a central cyclo­butene-1,3-dione ring.

## Spectroscopic Features   

The fully defined Cp′_3_U(BH_4_) compound was also characterized by ^1^H, ^11^B{^1^H}, ^13^C{^1^H}, and ^29^Si{^1^H} multi-nuclear NMR spectroscopy. It was of particular inter­est to examine the ^29^Si{^1^H} spectrum for comparison with previous studies of silicon-containing paramagnetic uranium complexes (Windorff & Evans, 2014 ▸). The ^1^H NMR spectrum in C_7_D_8_ was in good agreement with the literature (Berthet & Ephritikhine, 1992 ▸). ^11^B{^1^H}, ^13^C{^1^H}, and ^29^Si{^1^H} spectra were also obtained in both C_7_D_8_ and C_6_D_6_, as well as different field strengths, 500 *vs* 600 MHz for ^1^H, to see if any significant solvent or field effects were present. Since the spectra were not dependent on solvent or field strength, only the spectra obtained in C_6_D_6_ in a 600 MHz field will be discussed here. See Section 6 for full details.

In general, the resonances attributable to the Cp′^−^ ligands are sharp (*ν*_1/2_ < 50 Hz) and paramagnetically shifted over a range of *δ* 9.6 to −22.6 ppm, in the ^1^H NMR spectrum, and a ^29^Si{^1^H} resonance at *δ* −57.4 ppm was observed, typical of other tetra­valent uranium complexes (Windorff & Evans, 2014 ▸). The resonances attributable to the (BH_4_)^−^ unit showed considerably more shifting and broadening, resonating at *δ* −59.5 (*ν*_1/2_ = 300 Hz) and 79.6 (*ν*_1/2_ = 240 Hz) in the ^1^H and ^11^B{^1^H} spectra, respectively. Since the (BH_4_)^−^ ligand exhibited a single ^1^H NMR resonance whereas two distinct hydride environments are present in the solid state, it appears that the complex is fluxional in solution. This is in line with previous studies (Ephritikhine, 1997 ▸).

## Synthesis and crystallization   

### General considerations   

All manipulations and syntheses described below were conducted with the rigorous exclusion of air and water using glovebox techniques under an argon atmosphere. Solvents (THF, Et_2_O, toluene, hexane, and penta­ne) were sparged with UHP argon (Praxair) and dried by passage through columns containing a copper(II) oxide oxygen scavenger (Q-5) and mol­ecular sieves prior to use or stirred over sodium benzo­phenone ketyl, briefly exposed to vacuum several times to degas and distilled under vacuum. All ethereal solvents were stored over activated 4 Å mol­ecular sieves. Deuterated solvents (Cambridge Isotopes) used for nuclear magnetic resonance (NMR) spectroscopy were dried over sodium benzo­phenone ketyl, degassed by three freeze-pump-thaw cycles, and distilled under vacuum before use. The ^1^H, ^11^B{^1^H}, ^13^C{^1^H} and ^29^Si{^1^H} NMR spectra were recorded on a GN 500, Cryo 500 or Bruker Avance 600 spectrometer operating at 500.2 MHz, 160.1 MHz, 125.8 MHz, and 99.1 MHz for the 500 MHz spectrometers, respectively, and 600.1 MHz, 192.6 MHz, 150.9 MHz and 119.2 MHz for the 600 MHz spectrometer, respectively, at 298 K unless otherwise stated. The ^1^H and ^13^C{^1^H} NMR spectra were referenced inter­nally to solvent resonances, ^11^B and ^29^Si{^1^H} NMR spectra were referenced externally to BF_3_(Et_2_O) and SiMe_4_, respectively, the ^29^Si{^1^H} spectra were acquired using the INEPT pulse sequence. The 15-crown-5 (Aldrich) reagent was dried over activated mol­ecular sieves and degassed by three freeze–pump–thaw cycles before use. The NaBH_4_ (Aldrich) reagent was placed under vacuum (10 ^−3^ Torr) for 12 h before use. The following compounds were prepared following literature procedures: KCp′ (Peterson *et al.*, 2013 ▸), U(BH_4_)_4_ (Schles­inger & Brown, 1953 ▸), Cp′_3_UI (Windorff *et al.*, 2017 ▸).

### Cp′_3_U(BH_4_) from Cp′_3_UI, NaBH_4_ and 15-crown-5   

Solid NaBH_4_ (15 mg, 0.40 mmol) was added to a C_7_D_8_ (toluene-*d*_8_, 0.6 mL) solution of Cp′_3_UI (37 mg, 0.048 mmol) in a J-Young NMR tube, an excess of 15-crown-5 (1 drop) was added and the tube was sealed and removed from the glovebox and vortexed (30 s). The NaBH_4_ was not fully soluble in C_7_D_8_ even in the presence of 15-crown-5. After 18 h, NMR spectroscopy showed complete conversion to Cp′_3_U(BH_4_). The sample was brought back into the glovebox and the volatiles were removed under reduced pressure. The product was then extracted into Et_2_O, filtered away from white insoluble solids [presumably Na(15-crown-5)I and excess NaBH_4_] and the volatiles were removed under reduced pressure to give Cp′_3_U(BH_4_) (30 mg, 96%) as a wine-red solid. ^1^H NMR (C_7_D_8_, 500.2 MHz): *δ* 9.7 (*s*, C_5_ *H*_4_SiMe_3_, 6H), −2.1 (*s*, C_5_H_4_Si*Me*_3_, 27H), −23.1 (*s*, C_5_ *H*_4_SiMe_3_, 6H), −59.8 (*s*, *br*, *ν*_1/2_ = 325 Hz, U—(B*H*_4_), 4H); ^11^B{^1^H} NMR (C_7_D_8_, 160.1 MHz): *δ* 79.1 [*s*, *br*, *ν*_1/2_ = 230 Hz, U—(*B*H_4_)]; ^13^C{^1^H} NMR (C_7_D_8_, 125.8 MHz): *δ* 233.1 (*C*_5_H_4_SiMe_3_), 214.0 (*C*_5_H_4_SiMe_3_), 185.6 (*C*_5_H_4_SiMe_3_), 0.4 (C_5_H_4_Si*Me*_3_); ^29^Si{^1^H} NMR (C_7_D_8_, 99.1 MHz, INEPT): *δ* −57.7 (*s*, C_5_H_4_ *Si*Me_3_); ^1^H NMR (C_6_D_6_, 600.1 MHz): *δ* 9.6 (*s*, C_5_ *H*_4_SiMe_3_, 6H), −2.0 (*s*, C_5_H_4_Si*Me*_3_, 27H), −22.6 (*s*, C_5_ *H*_4_SiMe_3_, 6H), −59.3 (*s*, *br*, ν_1/2_ = 300 Hz, U—(B*H*_4_), 4H); ^11^B{^1^H} NMR (C_6_D_6_, 192.6 MHz): *δ* 79.6 [*s*, *br*, *ν*_1/2_ = 240 Hz, U-(*B*H_4_)]; ^13^C{^1^H} NMR (C_6_D_6_, 150.9 MHz): *δ* 232.0 (*C*_5_H_4_SiMe_3_), 214.2 (*C*_5_H_4_SiMe_3_), 186.5 (*C*_5_H_4_SiMe_3_), 0.6 (C_5_H_4_Si*Me*_3_); ^29^Si{^1^H} NMR (C_6_D_6_, 119.2 MHz, INEPT): *δ* −57.4 (*s*, C_5_H_4_ *Si*Me_3_).

### Cp′_3_U(BH_4_) from U(BH_4_)_4_ and KCp′   

An Et_2_O (5 mL) solution of KCp′ (460 mg, 2.61 mmol) was added to a pale-green solution of U(BH_4_)_4_ (250 mg, 0.841 mmol), also dissolved in Et_2_O (5 mL). White solids precipitated (presumably KBH_4_) as the solution quickly turned orange and then slowly changed to dark red (30 min). After stirring the mixture for an additional 12 h, volatiles were removed under reduced pressure, and the product was extracted into hexane leaving white solids behind (presumably KBH_4_). Removal of the volatiles under reduced pressure gave Cp′_3_U(BH_4_) (496 mg, 89%) as a dark wine-red solid. X-ray quality crystals were grown from a concentrated ether solution at 253 K.

## Refinement   

Crystal data, data collection and structure refinement details are summarized in Table 2 ▸. Analytical scattering factors neutral atoms were used throughout the analysis. A 3-D rendering of the mol­ecule can be found at the following web address: https://submission.iucr.org/jtkt/serve/z/Utgd9EjfTrqJVoXA/zz0000/0/.

C—H bond distances were constrained to 0.95 Å for cyclo­penta­dienyl C—H moieties, and to 0.98 Å for aliphatic CH_3_ moieties, respectively. Methyl torsion angles were not refined but constrained to be staggered. The borohydride H atoms were located from a difference-Fourier map and their positions were freely refined. *U*_iso_(H) values were set to a multiple of *U*_eq_(C/B) with 1.5 for CH_3_ and BH_4_ and 1.2 for C—H units, respectively.

## Supplementary Material

Crystal structure: contains datablock(s) I. DOI: 10.1107/S2056989021002425/zl5005sup1.cif

Structure factors: contains datablock(s) I. DOI: 10.1107/S2056989021002425/zl5005Isup3.hkl

CCDC reference: 2067867

Additional supporting information:  crystallographic information; 3D view; checkCIF report

## Figures and Tables

**Figure 3:**
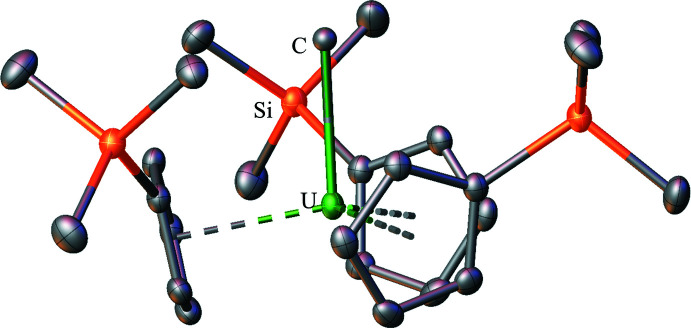
Structure of Cp′_3_UCH_3_/Cl with only the (CH_3_)^−^ ligand shown, with atomic displacement parameters drawn at the 50% probability level, except the –CH_3_ unit, which has been plotted as an isotropic sphere, as reproduced from the published CIF (Windorff *et al.*, 2017 ▸), see the manuscript for further details. Hydrogen atoms are omitted for clarity.

**Figure 4:**
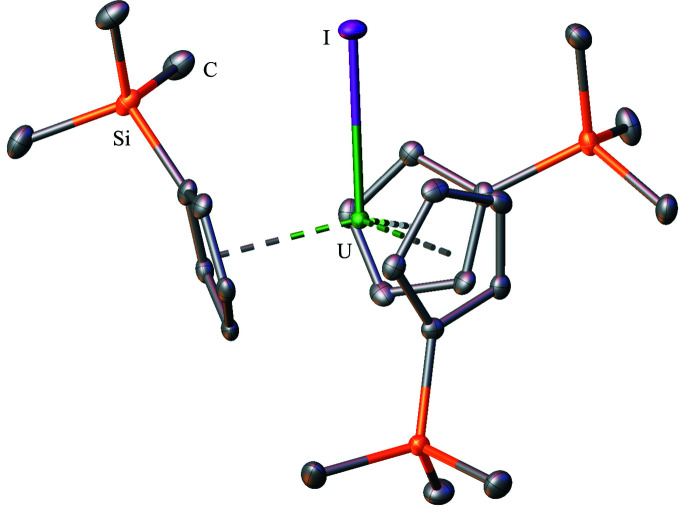
Structure of Cp′_3_UI with atomic displacement parameters drawn at the 50% probability level, as reproduced from the published CIF (Windorff *et al.*, 2017 ▸). Hydrogen atoms are omitted for clarity.

**Figure 5:**
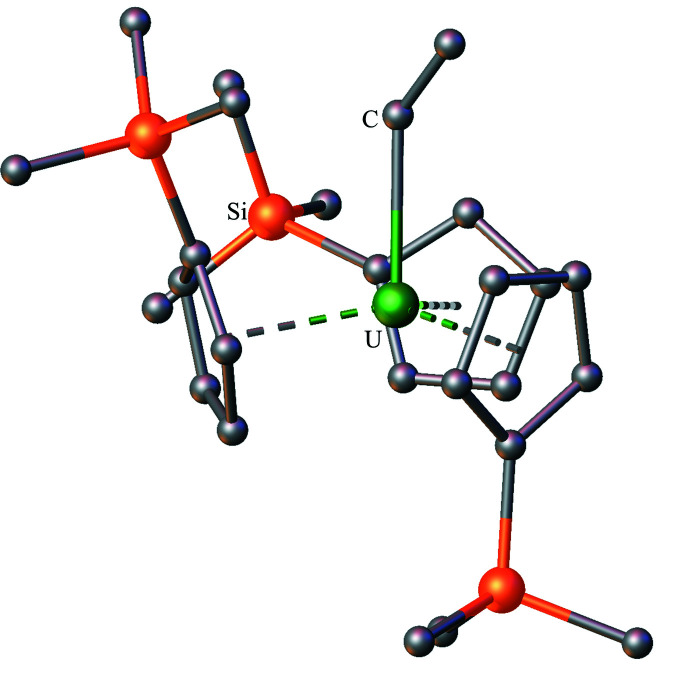
Structure of Cp′_3_U(*η* ^1^-CH=CH_2_) with atomic displacement parameters drawn as isotropic spheres, as reproduced from the CIF (Schock *et al.*, 1988 ▸). Hydrogen atoms are omitted for clarity.

**Figure 6:**
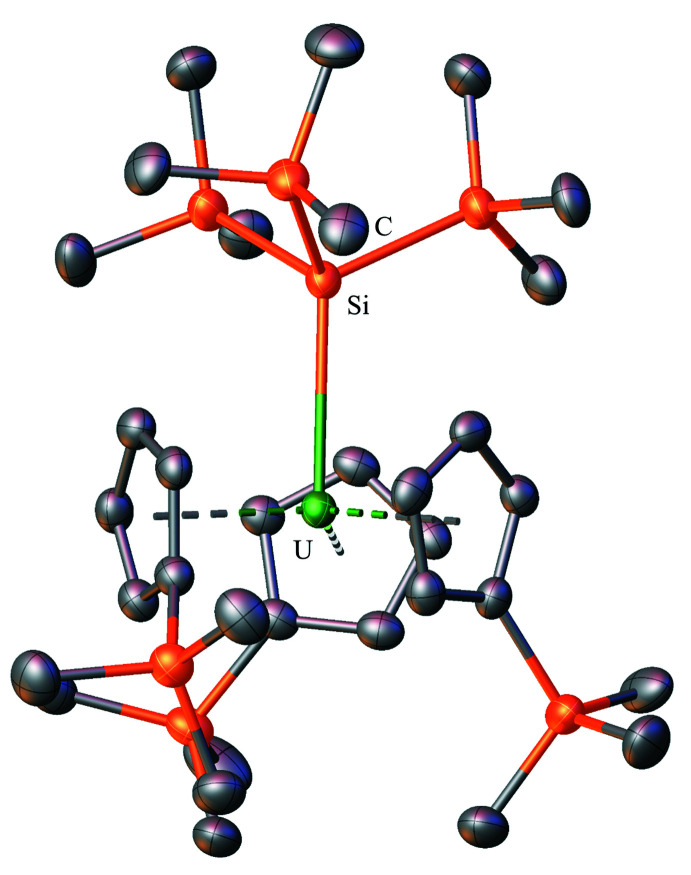
Structure of Cp′_3_U[Si(SiMe_3_)_3_] with atomic displacement parameters drawn at the 50% probability level, as reproduced from the published CIF (Réant *et al.*, 2020 ▸); the isomorphous thorium complex, Cp′_3_Th[Si(SiMe_3_)_3_], is also known (Réant *et al.*, 2020 ▸). Hydrogen atoms are omitted for clarity.

**Figure 7:**
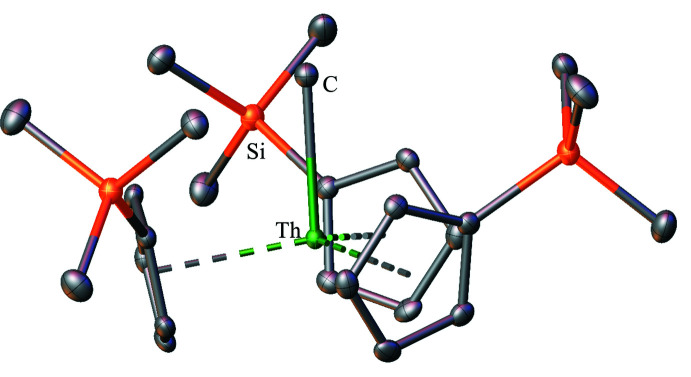
Structure of Cp′_3_ThCH_3_ with atomic displacement parameters drawn at the 50% probability level, as reproduced from the published CIF (Wedal *et al.*, 2019 ▸). Hydrogen atoms are omitted for clarity.

**Figure 8:**
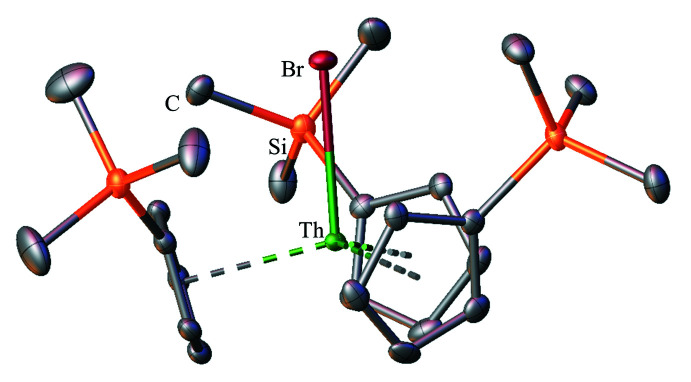
Structure of Cp′_3_ThBr with atomic displacement parameters drawn at the 50% probability level, as reproduced from the published CIF in the *P* 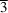 space group (Windorff *et al.*, 2017 ▸); there is a second report of the same mol­ecule in the *P*2_1_/*c* space group, featuring the same ligand orientation (Wedal *et al.*, 2019 ▸). Hydrogen atoms are omitted for clarity.

**Figure 9:**
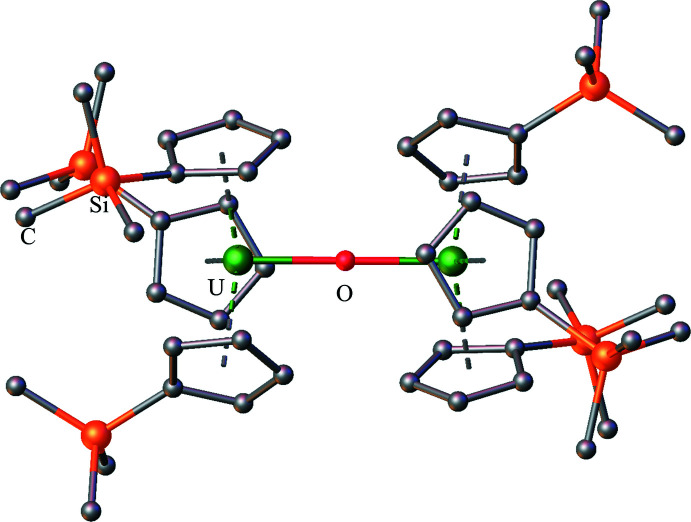
Structure of (Cp′_3_U)_2_(*μ*-O) with atomic displacement parameters drawn as isotropic spheres, as reproduced from the CIF (Berthet *et al.*, 1991 ▸); the isomorphous thorium complex, (Cp′_3_Th)_2_(*μ*-O), is also known (Wedal *et al.*, 2019 ▸). Hydrogen atoms are omitted for clarity.

**Figure 10:**
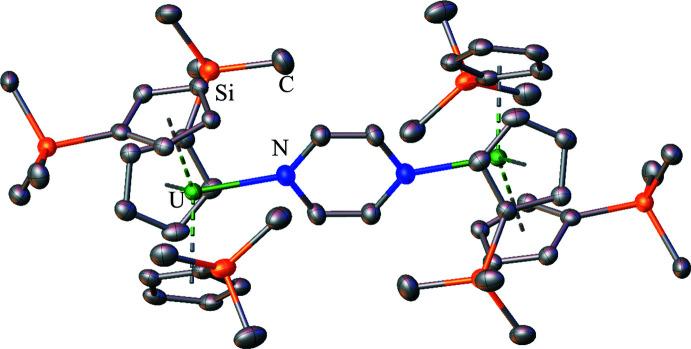
Structure of (Cp′_3_U)_2_[*μ*-(N_2_C_4_H_4_)] with atomic displacement parameters drawn at the 50% probability level, as reproduced from the published CIF (Mehdoui *et al.*, 2004 ▸). Hydrogen atoms are omitted for clarity.

**Figure 11:**
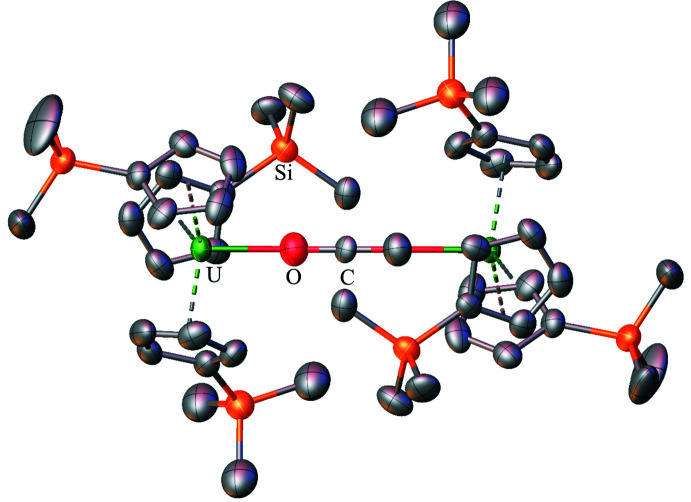
Structure of (Cp′_3_U)_2_(*μ*-CCO) with atomic displacement parameters drawn at the 50% probability level and disorder in the (*μ*-CCO)^2−^ unit displayed in one configuration, as reproduced from the published CIF (Tsoureas & Cloke, 2018 ▸). The mol­ecule contains a plane of symmetry, and the unit cell contains two half mol­ecules with the same orientation. For clarity, only one full mol­ecular unit is depicted and hydrogen atoms are omitted for clarity.

**Figure 12:**
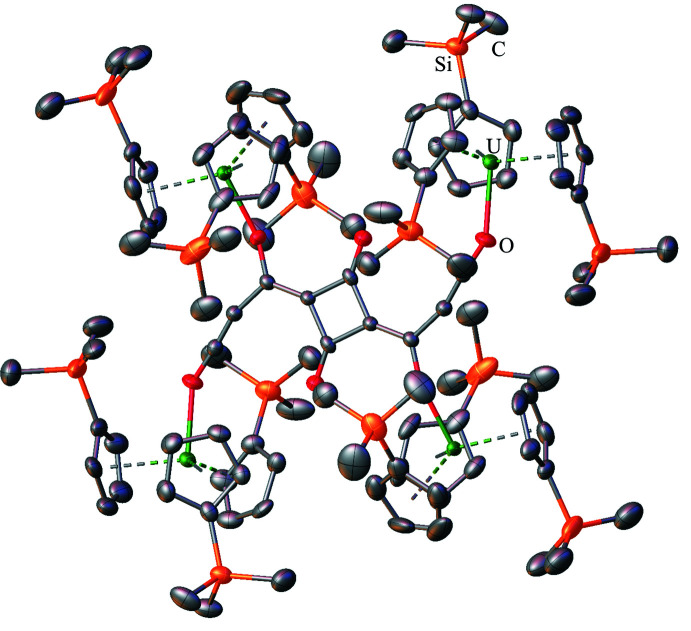
Structure of (Cp′_3_U)_4_(*μ*-*L*) where *L* = a complex organic structure containing a central cyclo­butene-1,3-dione ring, with atomic displacement parameters drawn at the 50% probability level, as reproduced from the published CIF (Tsoureas & Cloke, 2018 ▸). Hydrogen atoms and disorder in the –SiMe_3_ groups are omitted for clarity.

**Table 1:** A comparison of structural parameters (Å, °) in Cp′_3_U(BH_4_) and other Cp′_3_U*X* {*X* = Cl^−^, I^−^, [Si(SiMe_3_)_3_]^−^} complexes cent = C_5_H_4_SiMe_3_ centroid.

	Cp′_3_U(BH_4_)	Cp′_3_UCl*^*a*^* (Windorff *et al.*, 2017 ▸)	Cp′_3_UI (Windorff *et al.*, 2017 ▸)	Cp′_3_U(*η* ^1^-CH=CH_2_) (Schock *et al.*, 1988 ▸)	Cp′_3_U[Si(SiMe_3_)_3_] (Réant *et al.*, 2020 ▸)
U—(cent)	2.458, 2.490, 2.500	2.473	2.475, 2.478, 2.480	2.481, 2.483, 2.489	2.472, 2.478, 2.485
(cent)—U—*X*	104.13, 104.14, 104.83	100.00	97.9, 101.2, 101.6	95.1 100.0 100.2	96.04, 96.30, 97.65
(cent)—U—(cent)	113.28, 114.26, 114.26	117.00	116.1, 116.4, 118.3	116.4, 117.2, 120.0	118.28, 118.88, 119.08

Note: (*a*) The asymmetric unit contains one Cp′ ring, one-third of a chloride atom, and one-third of a uranium atom.

**Table 2:** Experimental details

Crystal data	
Chemical formula	[U(BH_4_)(C_8_H_13_Si)_3_]
*M*_r_	664.69
Crystal system, space group	Triclinic, *P*\overline{1}
Temperature (K)	112
*a*, *b*, *c* (Å)	8.7530 (15), 12.217 (2), 13.657 (2)
α, β, γ (°)	94.159 (3), 96.016 (3), 103.256 (3)
*V* (Å^3^)	1406.6 (4)
*Z*	2
Radiation type	Mo *K*α
μ (mm^−1^)	5.91
Crystal size (mm)	0.88 × 0.62 × 0.17

Data collection	
Diffractometer	Bruker D8 Quest with Photon II detector
Absorption correction	Multi-scan (*SADABS*; Krause *et al.*, 2015 ▸)
*T*_min_, *T*_max_	0.413, 0.747
No. of measured, independent and observed [*I* > 2σ(*I*)] reflections	30231, 10686, 9355
*R*_int_	0.055
(sin θ/λ)_max_ (Å^−1^)	0.769

Refinement	
*R*[*F* ^2^ > 2σ(*F* ^2^)], *wR*(*F* ^2^), *S*	0.037, 0.102, 1.07
No. of reflections	10686
No. of parameters	274
H-atom treatment	H atoms treated by a mixture of independent and constrained refinement
Δρ_max_, Δρ_min_ (e Å^−3^)	3.87, −2.72

Computer programs: *APEX3* and *SAINT* (Bruker, 2018 ▸), *SHELXS97* (Sheldrick, 2008 ▸), *SHELXL2018/3* (Sheldrick, 2015 ▸), and *SHELXTL2018/3* (Sheldrick, 2008 ▸).

## supplementary crystallographic information

### Crystal data

**Table:**

[U(BH_4_)(C_8_H_13_Si)_3_]	*Z* = 2
*M**_r_* = 664.69	*F*(000) = 652
Triclinic, *P*1	*D*_x_ = 1.569 Mg m^−^^3^
*a* = 8.7530 (15) Å	Mo *K*α radiation, λ = 0.71073 Å
*b* = 12.217 (2) Å	Cell parameters from 30231 reflections
*c* = 13.657 (2) Å	θ = 2.4–33.1°
α = 94.159 (3)°	µ = 5.91 mm^−^^1^
β = 96.016 (3)°	*T* = 112 K
γ = 103.256 (3)°	Plate, red
*V* = 1406.6 (4) Å^3^	0.88 × 0.62 × 0.17 mm

### Data collection

**Table:**

Bruker D8 Quest with Photon II detector diffractometer	9355 reflections with *I* > 2σ(*I*)
Radiation source: IµS 3.0 microfocus	*R*_int_ = 0.055
ω scans	θ_max_ = 33.1°, θ_min_ = 2.4°
Absorption correction: multi-scan (SADABS; Krause *et al.*, 2015)	*h* = −13→13
*T*_min_ = 0.413, *T*_max_ = 0.747	*k* = −18→18
30231 measured reflections	*l* = −20→20
10686 independent reflections	

### Refinement

**Table:**

Refinement on *F*^2^	Primary atom site location: structure-invariant direct methods
Least-squares matrix: full	Secondary atom site location: difference Fourier map
*R*[*F*^2^ > 2σ(*F*^2^)] = 0.037	Hydrogen site location: mixed
*wR*(*F*^2^) = 0.102	H atoms treated by a mixture of independent and constrained refinement
*S* = 1.07	*w* = 1/[σ^2^(*F*_o_^2^) + (0.0549*P*)^2^] where *P* = (*F*_o_^2^ + 2*F*_c_^2^)/3
10686 reflections	(Δ/σ)_max_ = 0.002
274 parameters	Δρ_max_ = 3.87 e Å^−^^3^
0 restraints	Δρ_min_ = −2.72 e Å^−^^3^

### Special details

**Table:**

Geometry. All esds (except the esd in the dihedral angle between two l.s. planes) are estimated using the full covariance matrix. The cell esds are taken into account individually in the estimation of esds in distances, angles and torsion angles; correlations between esds in cell parameters are only used when they are defined by crystal symmetry. An approximate (isotropic) treatment of cell esds is used for estimating esds involving l.s. planes.

### Fractional atomic coordinates and isotropic or equivalent isotropic displacement parameters (Å^2^)

**Table:**

	*x*	*y*	*z*	*U*_iso_*/*U*_eq_	
U1	0.51249 (2)	0.16808 (2)	0.26353 (2)	0.00963 (4)	
B1	0.8150 (5)	0.2329 (4)	0.3009 (4)	0.0177 (8)	
H1A	0.761 (6)	0.294 (5)	0.308 (4)	0.027*	
H1B	0.771 (6)	0.205 (5)	0.219 (4)	0.027*	
H1C	0.762 (6)	0.166 (5)	0.348 (4)	0.027*	
H1D	0.944 (6)	0.271 (5)	0.317 (4)	0.027*	
Si1	0.76504 (13)	0.39347 (9)	0.07862 (8)	0.0156 (2)	
Si2	0.71528 (13)	0.35432 (9)	0.54090 (9)	0.0161 (2)	
Si3	0.79753 (13)	−0.07373 (10)	0.23562 (8)	0.0161 (2)	
C1	0.5747 (4)	0.3269 (3)	0.1255 (3)	0.0134 (6)	
C2	0.4548 (4)	0.2353 (3)	0.0762 (3)	0.0142 (7)	
H2	0.467664	0.185642	0.022485	0.017*	
C3	0.3126 (4)	0.2291 (3)	0.1192 (3)	0.0168 (7)	
H3A	0.205686	0.182752	0.090235	0.020*	
C4	0.3423 (5)	0.3169 (3)	0.1952 (3)	0.0194 (8)	
H4A	0.259820	0.343109	0.229549	0.023*	
C5	0.5036 (5)	0.3754 (3)	0.2021 (3)	0.0170 (7)	
H5A	0.551554	0.449334	0.242324	0.020*	
C6	0.8516 (6)	0.2832 (4)	0.0182 (4)	0.0258 (9)	
H6A	0.773894	0.237713	−0.034755	0.039*	
H6B	0.946750	0.320007	−0.009698	0.039*	
H6C	0.879493	0.234315	0.067481	0.039*	
C7	0.9080 (5)	0.4833 (4)	0.1805 (4)	0.0259 (9)	
H7A	0.935861	0.435704	0.231009	0.039*	
H7B	1.003659	0.521095	0.153741	0.039*	
H7C	0.859331	0.540147	0.210342	0.039*	
C8	0.7126 (6)	0.4851 (4)	−0.0173 (4)	0.0284 (10)	
H8A	0.637627	0.438442	−0.070950	0.043*	
H8B	0.664218	0.541988	0.012768	0.043*	
H8C	0.808545	0.522937	−0.043833	0.043*	
C9	0.5445 (4)	0.2558 (3)	0.4617 (3)	0.0139 (6)	
C10	0.5031 (4)	0.1358 (3)	0.4589 (3)	0.0129 (6)	
H10A	0.566960	0.090586	0.496241	0.016*	
C11	0.3457 (4)	0.0938 (3)	0.4159 (3)	0.0148 (7)	
H11A	0.280490	0.015468	0.418243	0.018*	
C12	0.2877 (4)	0.1864 (3)	0.3859 (3)	0.0159 (7)	
H12A	0.174487	0.184244	0.363546	0.019*	
C13	0.4089 (5)	0.2856 (3)	0.4155 (3)	0.0156 (7)	
H13	0.400650	0.360371	0.406008	0.019*	
C14	0.7834 (5)	0.4881 (3)	0.4818 (4)	0.0236 (9)	
H14A	0.694252	0.523095	0.467930	0.035*	
H14B	0.867433	0.540488	0.526781	0.035*	
H14C	0.823995	0.470669	0.419794	0.035*	
C15	0.6386 (6)	0.3890 (4)	0.6594 (3)	0.0269 (9)	
H15A	0.553651	0.428013	0.645944	0.040*	
H15B	0.597529	0.319142	0.688904	0.040*	
H15C	0.724665	0.438154	0.705407	0.040*	
C16	0.8764 (5)	0.2804 (4)	0.5706 (3)	0.0226 (8)	
H16A	0.965381	0.332556	0.612059	0.034*	
H16B	0.836522	0.215057	0.606193	0.034*	
H16C	0.911947	0.254911	0.509097	0.034*	
C17	0.6012 (4)	−0.0360 (3)	0.2171 (3)	0.0128 (6)	
C18	0.4764 (5)	−0.0648 (3)	0.2755 (3)	0.0140 (6)	
H18	0.485999	−0.088223	0.340226	0.017*	
C19	0.3343 (4)	−0.0530 (3)	0.2215 (3)	0.0153 (7)	
H19	0.232617	−0.068713	0.243279	0.018*	
C20	0.3699 (4)	−0.0141 (3)	0.1306 (3)	0.0145 (7)	
H20A	0.290811	−0.013775	0.072251	0.017*	
C21	0.5350 (4)	−0.0017 (3)	0.1283 (3)	0.0130 (6)	
H21A	0.589324	0.009939	0.067662	0.016*	
C22	0.8854 (5)	−0.0487 (4)	0.3683 (3)	0.0238 (9)	
H22A	0.904791	0.031903	0.390761	0.036*	
H22B	0.811913	−0.092648	0.408388	0.036*	
H22C	0.985486	−0.072076	0.375288	0.036*	
C23	0.7622 (7)	−0.2283 (4)	0.1955 (4)	0.0334 (11)	
H23A	0.716059	−0.243268	0.125928	0.050*	
H23B	0.862889	−0.250933	0.203265	0.050*	
H23C	0.689316	−0.271505	0.236365	0.050*	
C24	0.9339 (5)	0.0070 (4)	0.1537 (4)	0.0277 (10)	
H24A	0.883975	−0.008159	0.084891	0.042*	
H24B	0.954902	0.088142	0.174393	0.042*	
H24C	1.033665	−0.016878	0.159288	0.042*	

### Atomic displacement parameters (Å^2^)

**Table:**

	*U*^11^	*U*^22^	*U*^33^	*U*^12^	*U*^13^	*U*^23^
U1	0.00980 (6)	0.01025 (6)	0.00983 (7)	0.00336 (4)	0.00194 (4)	0.00341 (4)
B1	0.0131 (18)	0.019 (2)	0.021 (2)	0.0041 (15)	0.0016 (16)	0.0059 (16)
Si1	0.0167 (5)	0.0131 (4)	0.0176 (5)	0.0025 (4)	0.0052 (4)	0.0052 (4)
Si2	0.0188 (5)	0.0123 (4)	0.0163 (5)	0.0030 (4)	0.0004 (4)	0.0002 (4)
Si3	0.0160 (5)	0.0195 (5)	0.0161 (5)	0.0099 (4)	0.0025 (4)	0.0049 (4)
C1	0.0143 (15)	0.0126 (15)	0.0135 (16)	0.0032 (12)	0.0010 (12)	0.0050 (12)
C2	0.0163 (16)	0.0158 (16)	0.0109 (16)	0.0038 (13)	0.0015 (13)	0.0037 (12)
C3	0.0140 (16)	0.0207 (18)	0.0172 (18)	0.0051 (13)	0.0010 (13)	0.0098 (14)
C4	0.0163 (17)	0.0192 (18)	0.028 (2)	0.0106 (14)	0.0061 (15)	0.0141 (16)
C5	0.0186 (17)	0.0148 (16)	0.0210 (19)	0.0079 (13)	0.0051 (14)	0.0091 (14)
C6	0.030 (2)	0.020 (2)	0.029 (2)	0.0048 (17)	0.0154 (19)	0.0026 (17)
C7	0.024 (2)	0.021 (2)	0.030 (2)	−0.0006 (16)	0.0056 (18)	0.0030 (17)
C8	0.028 (2)	0.029 (2)	0.032 (3)	0.0063 (18)	0.0100 (19)	0.021 (2)
C9	0.0145 (15)	0.0117 (15)	0.0165 (17)	0.0045 (12)	0.0024 (13)	0.0022 (12)
C10	0.0168 (16)	0.0122 (15)	0.0102 (15)	0.0033 (12)	0.0028 (12)	0.0028 (12)
C11	0.0148 (15)	0.0169 (16)	0.0132 (16)	0.0016 (13)	0.0061 (13)	0.0057 (13)
C12	0.0119 (15)	0.0236 (18)	0.0143 (17)	0.0067 (13)	0.0046 (13)	0.0026 (14)
C13	0.0167 (16)	0.0175 (17)	0.0145 (17)	0.0076 (13)	0.0025 (13)	0.0023 (13)
C14	0.026 (2)	0.0122 (17)	0.032 (2)	0.0031 (15)	0.0054 (18)	0.0032 (16)
C15	0.037 (2)	0.023 (2)	0.018 (2)	0.0031 (18)	0.0037 (18)	−0.0048 (16)
C16	0.0228 (19)	0.0204 (19)	0.025 (2)	0.0080 (15)	−0.0036 (16)	0.0027 (16)
C17	0.0141 (15)	0.0125 (15)	0.0126 (16)	0.0041 (12)	0.0025 (12)	0.0029 (12)
C18	0.0190 (17)	0.0126 (15)	0.0104 (16)	0.0033 (13)	0.0025 (13)	0.0024 (12)
C19	0.0137 (15)	0.0127 (15)	0.0182 (18)	−0.0003 (12)	0.0032 (13)	0.0031 (13)
C20	0.0140 (15)	0.0176 (16)	0.0097 (16)	0.0018 (13)	−0.0030 (12)	−0.0001 (12)
C21	0.0149 (16)	0.0140 (15)	0.0111 (16)	0.0063 (12)	0.0007 (12)	−0.0007 (12)
C22	0.0220 (19)	0.033 (2)	0.020 (2)	0.0142 (17)	0.0007 (16)	0.0059 (17)
C23	0.040 (3)	0.025 (2)	0.041 (3)	0.022 (2)	0.001 (2)	−0.001 (2)
C24	0.023 (2)	0.038 (3)	0.030 (2)	0.0164 (19)	0.0102 (18)	0.014 (2)

### Geometric parameters (Å, º)

**Table:**

U1—B1	2.568 (4)	C7—H7B	0.9800
U1—C21	2.731 (4)	C7—H7C	0.9800
U1—C10	2.732 (4)	C8—H8A	0.9800
U1—C20	2.733 (4)	C8—H8B	0.9800
U1—C5	2.740 (4)	C8—H8C	0.9800
U1—C12	2.748 (4)	C9—C13	1.419 (5)
U1—C11	2.754 (3)	C9—C10	1.424 (5)
U1—C4	2.755 (4)	C10—C11	1.403 (5)
U1—C3	2.755 (4)	C10—H10A	1.0000
U1—H1A	2.35 (5)	C11—C12	1.411 (5)
U1—H1B	2.35 (5)	C11—H11A	1.0000
U1—H1C	2.36 (5)	C12—C13	1.417 (5)
B1—H1A	0.98 (6)	C12—H12A	1.0000
B1—H1B	1.15 (6)	C13—H13	0.9500
B1—H1C	1.12 (5)	C14—H14A	0.9800
B1—H1D	1.11 (5)	C14—H14B	0.9800
Si1—C7	1.868 (5)	C14—H14C	0.9800
Si1—C8	1.871 (4)	C15—H15A	0.9800
Si1—C6	1.872 (5)	C15—H15B	0.9800
Si1—C1	1.877 (4)	C15—H15C	0.9800
Si2—C16	1.867 (4)	C16—H16A	0.9800
Si2—C9	1.871 (4)	C16—H16B	0.9800
Si2—C15	1.873 (5)	C16—H16C	0.9800
Si2—C14	1.877 (4)	C17—C18	1.415 (5)
Si3—C22	1.867 (5)	C17—C21	1.421 (5)
Si3—C23	1.873 (5)	C18—C19	1.420 (5)
Si3—C17	1.876 (4)	C18—H18	0.9500
Si3—C24	1.884 (5)	C19—C20	1.402 (5)
C1—C2	1.418 (5)	C19—H19	0.9500
C1—C5	1.432 (6)	C20—C21	1.421 (5)
C2—C3	1.420 (5)	C20—H20A	1.0000
C2—H2	0.9500	C21—H21A	1.0000
C3—C4	1.396 (6)	C22—H22A	0.9800
C3—H3A	1.0000	C22—H22B	0.9800
C4—C5	1.418 (5)	C22—H22C	0.9800
C4—H4A	1.0000	C23—H23A	0.9800
C5—H5A	1.0000	C23—H23B	0.9800
C6—H6A	0.9800	C23—H23C	0.9800
C6—H6B	0.9800	C24—H24A	0.9800
C6—H6C	0.9800	C24—H24B	0.9800
C7—H7A	0.9800	C24—H24C	0.9800
			
B1—U1—C21	91.29 (14)	C3—C4—H4A	125.3
B1—U1—C10	88.27 (13)	C5—C4—H4A	125.3
C21—U1—C10	121.25 (11)	U1—C4—H4A	125.3
B1—U1—C20	121.42 (14)	C4—C5—C1	108.8 (4)
C21—U1—C20	30.15 (11)	C4—C5—U1	75.7 (2)
C10—U1—C20	116.28 (11)	C1—C5—U1	77.6 (2)
B1—U1—C5	89.68 (13)	C4—C5—H5A	124.8
C21—U1—C5	119.23 (12)	C1—C5—H5A	124.8
C10—U1—C5	119.52 (12)	U1—C5—H5A	124.8
C20—U1—C5	115.78 (12)	Si1—C6—H6A	109.5
B1—U1—C12	128.78 (14)	Si1—C6—H6B	109.5
C21—U1—C12	131.96 (12)	H6A—C6—H6B	109.5
C10—U1—C12	48.97 (11)	Si1—C6—H6C	109.5
C20—U1—C12	104.45 (12)	H6A—C6—H6C	109.5
C5—U1—C12	89.90 (12)	H6B—C6—H6C	109.5
B1—U1—C11	117.73 (13)	Si1—C7—H7A	109.5
C21—U1—C11	113.80 (11)	Si1—C7—H7B	109.5
C10—U1—C11	29.63 (11)	H7A—C7—H7B	109.5
C20—U1—C11	95.57 (11)	Si1—C7—H7C	109.5
C5—U1—C11	118.89 (12)	H7A—C7—H7C	109.5
C12—U1—C11	29.73 (11)	H7B—C7—H7C	109.5
B1—U1—C4	119.54 (13)	Si1—C8—H8A	109.5
C21—U1—C4	115.96 (13)	Si1—C8—H8B	109.5
C10—U1—C4	114.79 (12)	H8A—C8—H8B	109.5
C20—U1—C4	98.00 (13)	Si1—C8—H8C	109.5
C5—U1—C4	29.91 (11)	H8A—C8—H8C	109.5
C12—U1—C4	70.43 (12)	H8B—C8—H8C	109.5
C11—U1—C4	99.66 (12)	C13—C9—C10	105.6 (3)
B1—U1—C3	129.05 (13)	C13—C9—Si2	125.9 (3)
C21—U1—C3	87.02 (12)	C10—C9—Si2	126.4 (3)
C10—U1—C3	134.56 (12)	C11—C10—C9	109.8 (3)
C20—U1—C3	69.70 (12)	C11—C10—U1	76.0 (2)
C5—U1—C3	49.08 (12)	C9—C10—U1	77.7 (2)
C12—U1—C3	85.59 (12)	C11—C10—H10A	124.4
C11—U1—C3	109.24 (11)	C9—C10—H10A	124.4
C4—U1—C3	29.34 (13)	U1—C10—H10A	124.4
B1—U1—H1A	22.4 (14)	C10—C11—C12	107.6 (3)
C21—U1—H1A	110.3 (14)	C10—C11—U1	74.3 (2)
C10—U1—H1A	88.4 (14)	C12—C11—U1	74.9 (2)
C20—U1—H1A	139.8 (14)	C10—C11—H11A	125.7
C5—U1—H1A	70.3 (13)	C12—C11—H11A	125.7
C12—U1—H1A	115.5 (14)	U1—C11—H11A	125.7
C11—U1—H1A	116.7 (14)	C11—C12—C13	107.6 (3)
C4—U1—H1A	99.3 (13)	C11—C12—U1	75.4 (2)
C3—U1—H1A	116.2 (13)	C13—C12—U1	76.6 (2)
B1—U1—H1B	26.5 (14)	C11—C12—H12A	125.4
C21—U1—H1B	70.8 (14)	C13—C12—H12A	125.4
C10—U1—H1B	113.0 (14)	U1—C12—H12A	125.4
C20—U1—H1B	100.0 (14)	C12—C13—C9	109.4 (3)
C5—U1—H1B	85.6 (13)	C12—C13—H13	125.3
C12—U1—H1B	154.6 (14)	C9—C13—H13	125.3
C11—U1—H1B	141.1 (13)	Si2—C14—H14A	109.5
C4—U1—H1B	113.1 (13)	Si2—C14—H14B	109.5
C3—U1—H1B	109.6 (13)	H14A—C14—H14B	109.5
H1A—U1—H1B	39.9 (19)	Si2—C14—H14C	109.5
B1—U1—H1C	25.8 (13)	H14A—C14—H14C	109.5
C21—U1—H1C	90.4 (14)	H14B—C14—H14C	109.5
C10—U1—H1C	66.9 (14)	Si2—C15—H15A	109.5
C20—U1—H1C	116.7 (13)	Si2—C15—H15B	109.5
C5—U1—H1C	112.4 (13)	H15A—C15—H15B	109.5
C12—U1—H1C	114.2 (14)	Si2—C15—H15C	109.5
C11—U1—H1C	94.8 (13)	H15A—C15—H15C	109.5
C4—U1—H1C	140.7 (13)	H15B—C15—H15C	109.5
C3—U1—H1C	154.7 (13)	Si2—C16—H16A	109.5
H1A—U1—H1C	42.1 (18)	Si2—C16—H16B	109.5
H1B—U1—H1C	46.4 (18)	H16A—C16—H16B	109.5
U1—B1—H1A	66 (3)	Si2—C16—H16C	109.5
U1—B1—H1B	66 (3)	H16A—C16—H16C	109.5
H1A—B1—H1B	97 (4)	H16B—C16—H16C	109.5
U1—B1—H1C	67 (3)	C18—C17—C21	106.5 (3)
H1A—B1—H1C	107 (4)	C18—C17—Si3	126.3 (3)
H1B—B1—H1C	110 (4)	C21—C17—Si3	125.2 (3)
U1—B1—H1D	174 (3)	C17—C18—C19	108.8 (3)
H1A—B1—H1D	108 (4)	C17—C18—H18	125.6
H1B—B1—H1D	115 (4)	C19—C18—H18	125.6
H1C—B1—H1D	118 (4)	C20—C19—C18	108.2 (3)
C7—Si1—C8	109.1 (2)	C20—C19—H19	125.9
C7—Si1—C6	111.5 (2)	C18—C19—H19	125.9
C8—Si1—C6	108.4 (2)	C19—C20—C21	107.5 (3)
C7—Si1—C1	110.74 (19)	C19—C20—U1	77.0 (2)
C8—Si1—C1	106.02 (19)	C21—C20—U1	74.8 (2)
C6—Si1—C1	110.83 (18)	C19—C20—H20A	125.5
C16—Si2—C9	109.96 (19)	C21—C20—H20A	125.5
C16—Si2—C15	107.7 (2)	U1—C20—H20A	125.5
C9—Si2—C15	105.7 (2)	C20—C21—C17	109.0 (3)
C16—Si2—C14	113.1 (2)	C20—C21—U1	75.0 (2)
C9—Si2—C14	111.05 (19)	C17—C21—U1	78.9 (2)
C15—Si2—C14	109.0 (2)	C20—C21—H21A	124.6
C22—Si3—C23	108.2 (2)	C17—C21—H21A	124.6
C22—Si3—C17	111.93 (19)	U1—C21—H21A	124.6
C23—Si3—C17	107.1 (2)	Si3—C22—H22A	109.5
C22—Si3—C24	111.8 (2)	Si3—C22—H22B	109.5
C23—Si3—C24	108.4 (3)	H22A—C22—H22B	109.5
C17—Si3—C24	109.23 (18)	Si3—C22—H22C	109.5
C2—C1—C5	105.6 (3)	H22A—C22—H22C	109.5
C2—C1—Si1	126.1 (3)	H22B—C22—H22C	109.5
C5—C1—Si1	126.4 (3)	Si3—C23—H23A	109.5
C1—C2—C3	109.7 (4)	Si3—C23—H23B	109.5
C1—C2—H2	125.2	H23A—C23—H23B	109.5
C3—C2—H2	125.2	Si3—C23—H23C	109.5
C4—C3—C2	107.5 (3)	H23A—C23—H23C	109.5
C4—C3—U1	75.3 (2)	H23B—C23—H23C	109.5
C2—C3—U1	75.9 (2)	Si3—C24—H24A	109.5
C4—C3—H3A	125.5	Si3—C24—H24B	109.5
C2—C3—H3A	125.5	H24A—C24—H24B	109.5
U1—C3—H3A	125.5	Si3—C24—H24C	109.5
C3—C4—C5	108.4 (4)	H24A—C24—H24C	109.5
C3—C4—U1	75.3 (2)	H24B—C24—H24C	109.5
C5—C4—U1	74.4 (2)		
			
C7—Si1—C1—C2	−164.8 (3)	Si2—C9—C10—U1	−128.1 (3)
C8—Si1—C1—C2	76.9 (4)	C9—C10—C11—C12	3.1 (4)
C6—Si1—C1—C2	−40.5 (4)	U1—C10—C11—C12	−68.1 (3)
C7—Si1—C1—C5	33.1 (4)	C9—C10—C11—U1	71.2 (3)
C8—Si1—C1—C5	−85.2 (4)	C10—C11—C12—C13	−2.8 (4)
C6—Si1—C1—C5	157.4 (3)	U1—C11—C12—C13	−70.5 (3)
C5—C1—C2—C3	1.5 (4)	C10—C11—C12—U1	67.7 (3)
Si1—C1—C2—C3	−163.6 (3)	C11—C12—C13—C9	1.6 (4)
C1—C2—C3—C4	0.3 (4)	U1—C12—C13—C9	−68.1 (3)
C1—C2—C3—U1	−69.1 (3)	C10—C9—C13—C12	0.3 (4)
C2—C3—C4—C5	−2.0 (4)	Si2—C9—C13—C12	−163.7 (3)
U1—C3—C4—C5	67.7 (3)	C22—Si3—C17—C18	−45.2 (4)
C2—C3—C4—U1	−69.7 (3)	C23—Si3—C17—C18	73.3 (4)
C3—C4—C5—C1	3.0 (4)	C24—Si3—C17—C18	−169.5 (4)
U1—C4—C5—C1	71.3 (3)	C22—Si3—C17—C21	153.2 (3)
C3—C4—C5—U1	−68.3 (3)	C23—Si3—C17—C21	−88.3 (4)
C2—C1—C5—C4	−2.8 (4)	C24—Si3—C17—C21	28.9 (4)
Si1—C1—C5—C4	162.3 (3)	C21—C17—C18—C19	2.5 (4)
C2—C1—C5—U1	67.2 (2)	Si3—C17—C18—C19	−161.9 (3)
Si1—C1—C5—U1	−127.7 (3)	C17—C18—C19—C20	−1.5 (4)
C16—Si2—C9—C13	−172.4 (3)	C18—C19—C20—C21	0.0 (4)
C15—Si2—C9—C13	71.6 (4)	C18—C19—C20—U1	−69.3 (3)
C14—Si2—C9—C13	−46.5 (4)	C19—C20—C21—C17	1.6 (4)
C16—Si2—C9—C10	26.8 (4)	U1—C20—C21—C17	72.3 (3)
C15—Si2—C9—C10	−89.2 (4)	C19—C20—C21—U1	−70.8 (3)
C14—Si2—C9—C10	152.7 (3)	C18—C17—C21—C20	−2.5 (4)
C13—C9—C10—C11	−2.1 (4)	Si3—C17—C21—C20	162.1 (3)
Si2—C9—C10—C11	161.8 (3)	C18—C17—C21—U1	67.2 (3)
C13—C9—C10—U1	68.0 (3)	Si3—C17—C21—U1	−128.1 (3)
